# A narrative review of nutritional components, health effects, and disease prevention mechanisms of dairy products

**DOI:** 10.3389/fpubh.2026.1799734

**Published:** 2026-04-08

**Authors:** Miao Wang, Qian Wang, Bin Chen, Jianguo Liu

**Affiliations:** 1 Key Laboratory of Microbial Resources and Drug Development of Guizhou Education Department, School of Stomatology, Zunyi Medical University, Zunyi, China; 2 Key Laboratory of Oral Disease Research of Guizhou Education Department, School of Stomatology, Zunyi Medical University, Zunyi, China

**Keywords:** dairy products, disease prevention, mechanisms of action, nutritional components, synergistic effects

## Abstract

As an integral component of the human diet, dairy products are rich in high-quality protein, lactoferrin, conjugated linoleic acid, calcium, vitamin D, and various other nutrients and bioactive compounds. They exert broad health benefits through core mechanisms involving multiple pathways and targets. This article systematically reviews the nutritional composition of dairy products, provides an in-depth analysis of their fundamental mechanisms—such as gut-immune axis regulation and antioxidant–anti-inflammatory synergy—and comprehensively summarizes their preventive value in metabolic-related diseases, bone health, degenerative diseases, and malignancies. The interplay and synergistic effects among multiple components and mechanisms are discussed, along with future research directions, to offer a scientific basis for the application of dairy products in public health and clinical nutrition.

## 1 Introduction

**Figure 1:**
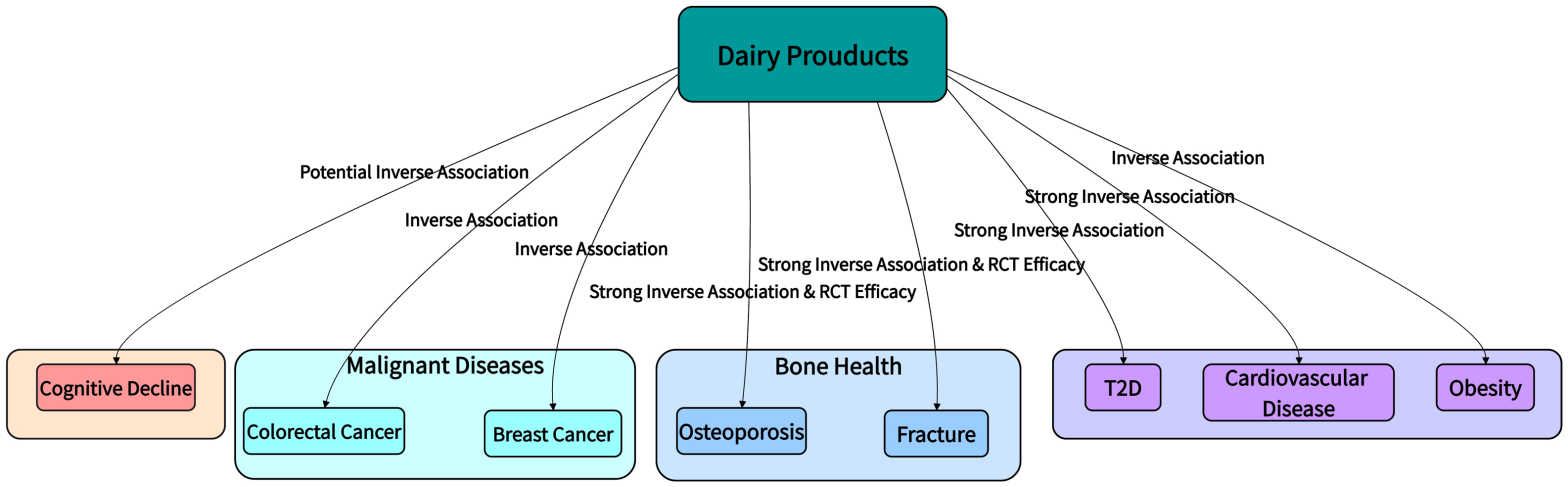
Overview of the association between dairy product intake and the risk of major chronic diseases. This figure summarizes the nature of epidemiological associations between dairy products and major chronic diseases. T2D, Type 2 diabetes.

Dairy products, encompassing a range of foods such as liquid milk, yogurt, cheese, and milk powder processed from raw materials like cow’s or goat’s milk, constitute a key component of global dietary patterns. With advancing research in nutritional science and public health, dairy products are no longer regarded merely as sources of energy and basic nutrients due to their rich content of high-quality protein, calcium, vitamin D, and probiotics. Instead, the significant role of their multiple bioactive components in preventing chronic non-communicable diseases and maintaining physiological homeostasis is increasingly recognized, securing their important place in human health maintenance. A substantial body of evidence from epidemiological investigations, clinical intervention trials, and mechanistic studies indicates that moderate dairy intake is associated with a reduced risk of developing various diseases, including type 2 diabetes, cardiovascular diseases, osteoporosis, and certain cancers (1–6), Figure 1 provides a systematic visual summary of the epidemiological associations between dairy product intake and the risk of major chronic diseases, laying a macro-evidence foundation for subsequent mechanism analysis and effect verification. However, the functional specificity of different dairy components, the synergy of their action mechanisms, and the heterogeneity of their effects across populations require systematic elucidation. Based on current research evidence, this review comprehensively examines the health value of dairy products across four dimensions: nutritional components, core mechanisms, disease-preventive effects, and synergistic interactions, aiming to inform the development of scientific dietary guidelines and clinical nutrition interventions.

This review strictly adheres to the methodological transparency standards for narrative reviews as required by the *Frontiers* series journals (detailed in Section 2). It provides a thorough overview of the disease prevention mechanisms and current research landscape related to dairy products, with a particular focus on core pathways such as gut microbiota modulation, anti-inflammatory and antioxidant effects, and metabolic improvement. By integrating findings from epidemiological surveys, clinical trials, and molecular biology research, this study critically evaluates the potential of dairy products in mitigating chronic disease risk and clearly distinguishes the evidence hierarchy among *in vitro* experiments, animal models, and human studies. The ultimate objective is to offer a robust theoretical foundation for shaping public health strategies and revising dietary guidelines, thereby contributing to reduced disease burden and enhanced population health.

## 2 Review methods

This review was conducted in strict accordance with the methodological transparency standards for narrative reviews outlined by *Frontiers* journals, employing a systematic evidence synthesis process. The specific steps are detailed below.

### 2.1 Search strategy

A combination of electronic database searches and manual searches was employed. Electronic searches covered three major international authoritative databases: PubMed, Web of Science Core Collection, and Scopus. The search timeframe was restricted from January 1, 2010, to March 31, 2025, to ensure the inclusion of both recent findings and seminal literature from the past 15 years. Core search terms were categorized and combined by research theme, primarily including: ① dairy-related terms (e.g., “dairy products,” “milk,” “yogurt,” “cheese,” “whey protein,” “lactoferrin,” “casein”); ② health outcome and mechanism-related terms (e.g., “health effects,” “chronic disease,” “antiviral,” “bone health,” “cardiovascular disease,” “gut microbiota,” “inflammation,” “oxidative stress”). Search strings were constructed using Boolean operators “AND” and “OR.” Examples include: “dairy products AND bone health AND randomized controlled trial” and “lactoferrin AND antiviral mechanism AND in vitro.” Additionally, the reference lists of key review articles were manually searched to supplement potentially missed important studies. Grey literature (e.g., unpublished research reports, conference abstracts) was not included due to limitations in methodological rigor and accessibility.

### 2.2 Inclusion and exclusion criteria

#### 2.2.1 Inclusion criteria

① Study type: Original research published in English, encompassing human clinical studies (randomized controlled trials, cohort studies, case–control studies, cross-sectional studies), animal experiments (involving model organisms such as mice, rats, rabbits), and *in vitro* mechanistic studies (cell culture, molecular biology experiments). ② Study content: Focused on the health effects, action mechanisms, or nutritional value of dairy products or their bioactive components (e.g., lactoferrin, whey protein, probiotics). ③ Quality standards: Studies with a complete design, detailed data, and clear conclusions.

#### 2.2.2 Exclusion criteria

① Publication type: Review articles, conference abstracts, dissertations/theses, letters, and duplicate publications. ② Research topic: Studies unrelated to the health effects of dairy products (e.g., focusing on dairy processing technology or sensory qualities) or solely concerned with non-health-related outcomes. ③ Data integrity: Studies with major design flaws, missing key data, or from which valid information could not be extracted.

### 2.3 Literature screening and evidence evaluation process

Literature screening was performed independently by two investigators, strictly following a two-step process: “title/abstract screening → full-text screening.” First, after obtaining records via search strings, both investigators reviewed titles and abstracts to preliminarily exclude records clearly not meeting the inclusion criteria. Second, the full texts of provisionally included records were retrieved and examined to verify whether all inclusion criteria were met, definitively excluding those that did not qualify. Any disagreements during screening were resolved through discussion and consensus involving a third investigator, with the points of disagreement and final decisions documented.

Study quality assessment employed a categorized approach: ① For human clinical studies: The risk of bias was assessed using the relevant Joanna Briggs Institute (JBI) critical appraisal tools (e.g., the JBI checklist for RCTs, the JBI checklist for cohort studies). ② For animal experiments: The Systematic Review Centre for Laboratory Animal Experimentation (SYRCLE) risk of bias tool was used. ③ For *in vitro* studies: The focus was on checking the rationale of the experimental design, the appropriateness of control groups, and replication. The strength of evidence was graded using the Grading of Recommendations, Assessment, Development, and Evaluations (GRADE) framework, categorizing the quality of evidence from included studies as High, Moderate, Low, or Very Low—where High indicates very high confidence in the estimated effect, and Very Low indicates that the true effect is likely to be substantially different from the estimate, warranting cautious interpretation.

## 3 Nutritional components of dairy products

**Figure 2:**
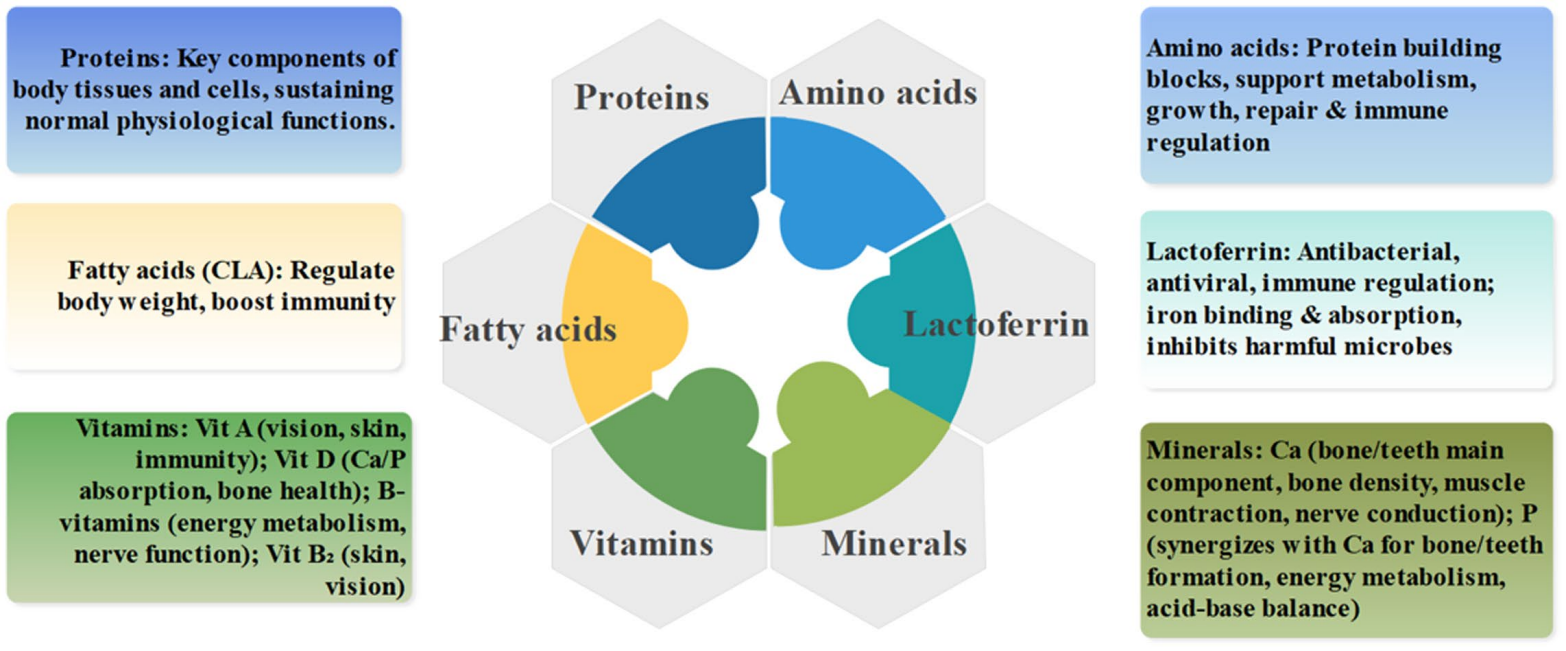
Overview of core nutritional and bioactive components of dairy products and their functions.

The health effects of dairy products stem from their complex and balanced composition of nutritional and bioactive components, which together form the material basis for their health benefits (7, 8). From a macro perspective, the core components of dairy products can be categorized into proteins, fatty acids, vitamins, minerals, amino acids, and lactoferrin, each with distinct functions that work synergistically (see Figure 2).

The following sections will elaborate on the specific composition and physiological functions of each category of components.

### 3.1 Proteins and amino acids

Dairy products serve as an important dietary source of high-quality protein, with common varieties such as milk, yogurt, and cheese being rich in protein of high biological value (7, 9). This protein contains all essential amino acids required by humans, with an amino acid profile that closely matches physiological needs. Its bioavailability ranks among the highest of natural food sources, allowing it to directly participate in core physiological processes such as cellular growth and repair, metabolic regulation, and immune function maintenance (9, 10). The FAO/WHO 2007 technical report explicitly states that the amino acid pattern of dairy protein fully meets human requirements across all life stages, establishing it as a key reference standard for high-quality protein (8).

As the foundational material for the immune system, dairy protein supplies the essential amino acids necessary for the proliferation and differentiation of immune cells, playing a significant role in maintaining immune homeostasis (10, 11). Notably, milk is particularly rich in lysine, an essential amino acid that serves as a crucial nutrient not only for growth and development in children but also for tissue repair and normal physiological function maintenance in adults (9). In a critical analysis of the 2007 WHO/FAO/UNU protein requirement report, Millward et al. (9) emphasized that the balanced ratio of lysine to tryptophan in dairy products is a core reason for their superior biological value compared to plant proteins.

Milk protein is primarily composed of casein (approximately 80%) and whey protein (approximately 20%) (12). Casein, a phospho-calcium binding protein, forms stable casein micelles through its quaternary structure in complex with calcium phosphate. This structure results in a slow digestion rate in the human gastrointestinal tract, enabling sustained release of amino acids and providing long-lasting nutritional support (11, 13). The casein composition and functional properties of proteins vary across different milk sources, such as bovine, caprine, and buffalo milk (14). Holt et al. (12) confirmed through protein chemistry research that the phosphorylation sites and calcium-binding capacity of casein directly influence its digestion and absorption rate, as well as mineral transport efficiency. In contrast, whey protein is characterized by rapid digestion and absorption. It is rich in bioactive components such as immunoglobulin G (IgG) and lactoferrin, and its content of branched-chain amino acids (leucine, isoleucine, and valine) is significantly higher than that of most other food proteins (10, 15). Bounous and Gold (10) found that the cysteine in whey protein can serve as a precursor for glutathione synthesis, thereby indirectly participating in the body’s antioxidant defense system. The cysteine-rich whey protein can serve as a precursor for the synthesis of glutathione, a crucial intracellular antioxidant (16).

Branched-chain amino acids can significantly promote muscle protein synthesis by activating the mTOR signaling pathway in skeletal muscle, playing a key role in muscle growth and post-exercise recovery (15, 17). A randomized crossover trial by Tatiana et al. (17) confirmed that supplementation with whey protein hydrolysate increased amino acid uptake in skeletal muscle by 30% and significantly elevated mTORC1 signaling pathway activation in healthy young men. For instance, athletes consuming dairy products rich in milk protein post-exercise can effectively accelerate muscle strength recovery and reduce exercise-induced muscle fatigue (15). A human intervention study by Cockburn et al. (15) showed that post-exercise intake of milk protein reduced levels of the muscle damage marker creatine kinase by 27% compared to a placebo group. However, it is important to note objectively that clinical evidence for this effect is primarily derived from *in vitro* and animal studies; large-scale randomized controlled trials confirming its generalizability in humans remain relatively scarce (13).

### 3.2 Characteristic bioactive proteins

#### 3.2.1 Lactoferrin

Lactoferrin is a distinctive multifunctional non-heme iron-binding glycoprotein found in dairy products. It is present at the highest concentration in human breast milk (1–3 mg/mL) and is also consistently found in dairy products such as bovine milk, yogurt, and cheese (0.02–0.3 mg/mL). It possesses multiple critical physiological functions, including antibacterial, antiviral, and immunomodulatory activities (18–20). A systematic review by Valenti and Antonini (19) clearly established that the iron-binding capacity of lactoferrin is closely related to its bioactivity, with apo-lactoferrin (iron-free) exhibiting significantly higher antibacterial and antiviral activity than holo-lactoferrin (iron-saturated).

In terms of its antibacterial action, lactoferrin can deprive pathogenic bacteria, such as *E. coli* and Salmonella, of the essential iron required for their growth and proliferation by chelating environmental iron ions with high affinity, thereby inhibiting their multiplication (19, 21). *In vitro* experiments by Ellison and Giehl (22) confirmed that lactoferrin exerts significant inhibitory effects on Gram-negative bacteria through a dual mechanism involving iron chelation and disruption of bacterial cell membranes. In the field of antiviral activity, its primary mechanism involves interfering with the binding of viruses to receptors on the surface of host cells, blocking the initial step of viral entry, and consequently reducing the risk of human cell infection (23, 24). Research by van der Strate et al. (24) indicates that lactoferrin inhibits viruses such as influenza and herpes viruses, with its blocking effect being most pronounced during the viral adsorption stage. It is important to note, however, that the antiviral effects of lactoferrin have primarily been validated in *in vitro* studies (e.g., inhibiting virus entry into Vero cells) and animal models (e.g., reducing influenza viral load in mice). Clinical evidence supporting a definitive antiviral role in humans remains relatively limited.

Regarding nutrient absorption, lactoferrin can bind to iron ions within the human intestinal tract to form soluble complexes, significantly enhancing the bioavailability of iron, which holds positive implications for preventing iron-deficiency anemia (25). A randomized, double-blind, placebo-controlled trial in healthy older adult women demonstrated that daily lactoferrin supplementation significantly upregulates the expression of the intestinal tight junction protein occludin, while simultaneously increasing the abundance of beneficial bacteria like Bifidobacterium, thereby strengthening intestinal barrier function and immune homeostasis (26). Furthermore, lactoferrin modulates the body’s immune function through various pathways, including activating the complement system and promoting the secretion of immune cytokines, effectively enhancing the response efficiency of both innate and adaptive immunity (27, 28). The research by Damiens et al. (28) found that lactoferrin can significantly augment the cytotoxic activity of natural killer (NK) cells against tumor cells.

#### 3.2.2 Other bioactive proteins

In addition to lactoferrin, dairy products contain various other bioactive proteins, including lactoperoxidase, lysozyme, and immunoglobulins (21, 24, 29). Lactoperoxidase helps maintain intestinal mucosal barrier integrity and reduce intestinal inflammation by generating hypochlorous acid (24); the review by Eker et al. (21) summarized the antibacterial mechanism of the lactoperoxidase system (lactoperoxidase-thiocyanate-hydrogen peroxide), confirming its inhibitory effects against multiple foodborne pathogens. Lysozyme exerts antibacterial action synergistically with lactoferrin by disrupting the peptidoglycan structure of bacterial cell walls (24); immunoglobulins, particularly IgG, can enhance intestinal immunity in infants and young children through a passive immune mechanism, thereby reducing the risk of infectious diarrhea (20, 24). Research by Jenssen and Hancock (29) indicates that the bioactive proteins in dairy products can form an antimicrobial network through synergistic interactions, with their combined effect being significantly superior to that of any single component. These bioactive proteins collectively constitute an important functional network responsible for the antimicrobial, antiviral, and immunomodulatory properties of dairy products.

### 3.3 Lipid-based bioactive components

The lipids in dairy products are far from being merely an energy source; they encompass a variety of bioactive components, including conjugated linoleic acid (CLA), the milk fat globule membrane (MFGM), and short-chain fatty acids (SCFAs) (30–33). CLA is a ruminant-specific fatty acid present in relatively high concentrations in milk and cheese (0.5–1.5 g/100 g fat). By activating the peroxisome proliferator-activated receptor (PPAR) pathway, it promotes hepatic lipid oxidation and metabolism, reduces triglyceride levels, and thereby decreases the risk of atherosclerosis (30, 32). A cell-based experiment by Shechter et al. (31) confirmed that CLA upregulates the gene expression of hepatic LDL receptors, promoting cholesterol clearance. Concurrently, CLA possesses the ability to inhibit the proliferation of tumor cells. *In vitro* experiments and animal studies have confirmed its significant inhibitory effects on the growth of tumor cells such as those from breast and colorectal cancers (31, 32, 34).

The milk fat globule membrane (MFGM) is a complex lipid membrane structure enveloping the milk fat globules. It is rich in bioactive components such as sphingomyelin, cholesterol, and glycerophospholipids. This structure not only helps regulate the composition of the gut microbiota and inhibits the proliferation of harmful bacteria but also promotes the formation of neural myelin. Consequently, it plays a positive role in both infant brain development and the maintenance of cognitive function in the older adults (30, 34, 35). As noted in a review by Camfield et al. (34), sphingomyelin within the MFGM can modulate neural cell signaling pathways, thereby improving cognitive function. Short-chain fatty acids (SCFAs, such as butyrate and acetate) produced during the fermentation of dairy products like yogurt and kefir can activate the GPR41/GPR43 receptors on intestinal epithelial cells. This activation contributes to the regulation of the body’s glucose metabolism by inhibiting the proliferation of colon cancer cells on one hand and improving insulin sensitivity on the other (32, 33).

### 3.4 Micronutrients and minerals

Dairy products are a natural dietary source high in calcium, with a content of approximately 100–120 mg per 100 mL. As a primary constituent of bones and teeth, calcium is crucial for maintaining skeletal health and structural integrity (35, 36). During human growth and development, adequate calcium intake forms the foundation for normal bone mineralization. Vitamin D, meanwhile, optimizes the efficiency of bone calcium deposition and significantly enhances calcium bioavailability by promoting intestinal calcium absorption and regulating renal calcium excretion (36). The National Osteoporosis Foundation’s (NOF) 2023 report explicitly identifies the synergistic action of calcium and vitamin D as a core dietary strategy for osteoporosis prevention (35).

A cluster randomized controlled trial by Iuliano et al. (37) confirmed that dietary fortification with dairy products (increasing calcium and protein intake) reduced the incidence of hip fractures by 30% among older adult residents in care homes. This synergistic effect between calcium and vitamin D positions dairy products as an ideal natural food for osteoporosis prevention (36, 38, 39). A review by Ratajczak et al. (40), analyzing multiple cohort studies, indicated that dairy intake is significantly inversely associated with the risk of osteoporotic fractures, a relationship particularly pronounced in older adults. Furthermore, dairy products contain various other essential micronutrients, including magnesium, selenium, and B vitamins (41–43). Magnesium, serving as a vital cofactor for glucokinase, enhances the phosphorylation efficiency of glucose in hepatocytes, thereby alleviating insulin resistance (42), which is crucial for maintaining systemic glucose homeostasis. Selenium contributes to the synthesis of glutathione peroxidase (GPx), bolstering the body’s antioxidant capacity and reducing oxidative damage to cells caused by reactive oxygen species (ROS) (41, 43), while also regulating the differentiation and function of immune cells. The combined action of vitamin D and calcium not only promotes bone mineralization and helps prevent osteoporosis (36, 39) but also optimizes the insulin-secretory function of pancreatic β-cells, thereby reducing the risk of diabetes (44, 45).

### 3.5 Other functional components

Probiotics (such as those from the Lactobacillus and Bifidobacterium genera) present in fermented dairy products are important functional constituents. They can modulate gut microbial balance, enhance intestinal barrier function, and regulate immune responses (46, 47). Beyond bovine milk, certain specialty milks like buffalo milk are also regarded as natural immunoadjuvants due to their unique compositional profiles (48). A review by Kerry et al. (47) summarized the multifaceted health effects of probiotics, including gut microbiota modulation, immune enhancement, and metabolic improvement. Prebiotics in dairy products, such as galactooligosaccharides (GOS) and fructooligosaccharides, promote the proliferation of probiotics and the subsequent production of SCFAs, further reinforcing gut health and glucose metabolism regulation (49, 50). Research by Mansilla and Sojo (51) confirmed that GOS can improve intestinal barrier function in patients with irritable bowel syndrome by modulating gut microbiota composition. Additionally, nucleotides found in dairy products can promote intestinal mucosal repair and enhance immune cell activity, making them particularly suitable for special population groups such as infants, young children, and older adults (50, 52). A review by Singh et al. (52) indicated that nucleotides in dairy products may reduce the risk of infectious diseases by regulating intestinal mucosal immunity. Furthermore, the functionality and activity of milk-derived bioactive peptides can be influenced by factors such as the lactating animal species (53).

**Figure 3:**
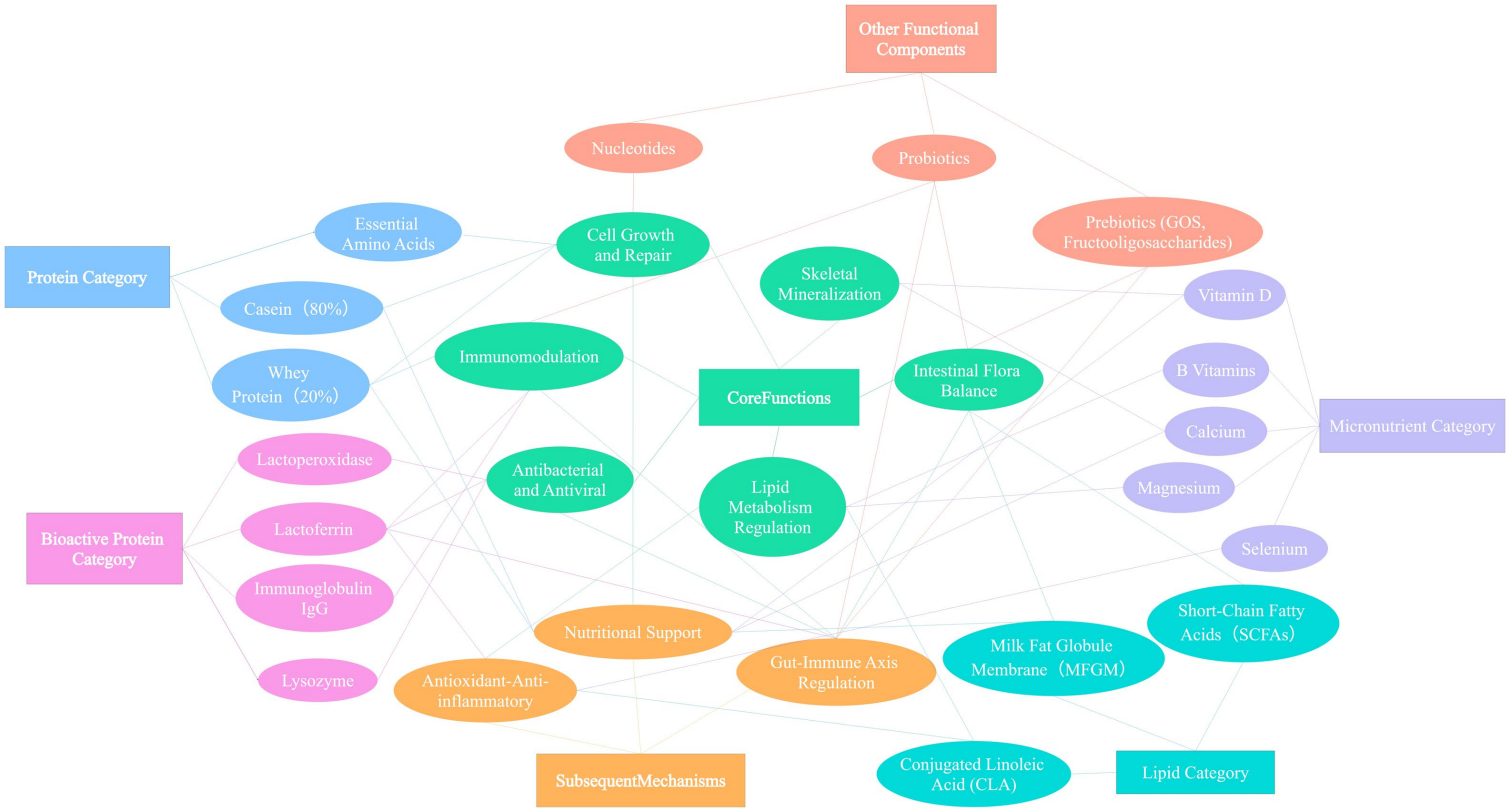
Classification and functional correlation diagram of nutritional components and bioactive substances in dairy products. This figure systematically combs the classification categories of core nutritional components (protein, calcium, vitamins, etc.) and characteristic bioactive substances (bioactive peptides, lactoferrin, probiotics, etc.) in dairy products, and intuitively presents the internal correlation and corresponding physiological functions of the two types of substances (such as immune regulation, bone health, intestinal maintenance, etc.). The classification logic and functional interpretation in the figure are all based on the confirmed research conclusions in the previous references (Nos. 1–98), providing a visual theoretical framework for elaborating on the health value of dairy products in the full text, and forming a response to the research direction of the subsequent references.

In addition to cow’s milk, special dairy sources such as goat milk, yak milk, and camel milk have attracted increasing attention due to their unique composition and functional properties. Goat milk has smaller fat globule diameter and looser casein micelle structure, leading to higher digestibility, making it suitable for populations with weak digestive function (54). Yak milk, produced under high-altitude and strong ultraviolet exposure, contains significantly higher fat, total protein, and polyphenols than conventional cow’s milk, exhibiting strong antioxidant and anti-inflammatory potential (55–57). Camel milk is rich in lactoferrin, lysozyme, immunoglobulins, and bioactive peptides, with potential advantages in regulating immune function and assisting glucose metabolism (58). Distinct differences exist among these milk sources in protein composition, fatty acid profile, milk oligosaccharide structure, and mineral content, providing a material basis for developing differentiated dairy products targeted at specific populations (54–58) (Figure 3).

## 4 Core fundamental mechanisms underlying the health effects of dairy products

The diverse health benefits of dairy products cannot be attributed to the independent action of any single component. Instead, they are primarily mediated through a set of core fundamental mechanisms—including nutritional support, gut-immune axis regulation, and antioxidant–anti-inflammatory synergy. These mechanisms collectively form a multi-pathway, multi-target regulatory network, providing the scientific foundation for their subsequent disease-preventive effects (8, 11, 30).

### 4.1 Providing nutritional support and promoting nutritional balance

The high-quality protein in dairy products contains all essential amino acids and exhibits high digestibility and absorption rates, supplying ample building blocks for bodily growth, development, and tissue repair (8–10). Specifically, whey protein hydrolysate promotes amino acid uptake in muscle tissue and activates the mTORC1 signaling pathway, demonstrating significant efficacy in maintaining muscle mass and enhancing post-exercise recovery (17). Minerals such as calcium, vitamin D, and magnesium, in synergy with vitamins, ensure the proper functioning of physiological processes including bone metabolism, nerve conduction, and muscle contraction (36, 39). Concurrently, they bolster the body’s immune defense capabilities by modulating the secretion levels of immune cell cytokines (11).

### 4.2 Gut-immune axis regulatory mechanisms

The gut is the largest immune organ in the body. Dairy products contribute to a core regulatory network of the gut-immune axis by modulating gut microbiota balance and reinforcing intestinal mucosal barrier function (49, 50, 59). As noted in a review by Odenwald and Turner (59), the integrity of the intestinal mucosal barrier is closely linked to gut-immune axis function, and dairy components can preserve this barrier through multiple pathways. Probiotics in dairy products can selectively colonize the intestine, inhibiting the growth of harmful bacteria such as *E. coli* and Salmonella while promoting the proliferation of beneficial bacteria like Bifidobacterium and Lactobacillus, thereby optimizing the gut microbial structure (46, 47). Simultaneously, short-chain fatty acids (SCFAs) produced by probiotic fermentation lower intestinal pH, further suppressing pathogen growth. Additionally, SCFAs serve as an energy source for intestinal epithelial cells, promoting the proliferation and repair of mucosal cells and thereby enhancing the integrity of the intestinal mucosal barrier (33, 50).

Strengthening intestinal mucosal barrier function reduces the entry of incompletely digested macromolecules, allergens, and pathogens into the bloodstream, lowering the risk of systemic inflammation (20, 60). An animal study by Li et al. (61) confirmed that casein-derived peptides could modulate gut microbiota composition, upregulate the expression of intestinal tight junction proteins (occludin and ZO-1), and reduce intestinal permeability. Furthermore, gut dysbiosis is closely associated with immune dysfunction. By regulating gut microbiota composition, dairy products can modulate the activity of immune cells (e.g., T lymphocytes and macrophages), promote the secretion of anti-inflammatory cytokines (e.g., IL-10), and inhibit the production of pro-inflammatory cytokines (e.g., TNF-α and IL-6), thereby helping to maintain immune homeostasis (27, 62). Research by Calder (62) emphasizes that the modulation of the gut-immune axis by dairy products holds significant importance in preventing inflammation-related diseases.

### 4.3 Synergistic effects of antioxidant and anti-inflammatory functions

Oxidative stress and chronic inflammation are common pathological foundations for the development of various chronic diseases, such as cardiovascular diseases, cancer, and metabolic syndrome (30, 41, 43). As noted in a review by Gombart et al. (43), the synergistic antioxidant–anti-inflammatory action of micronutrients and bioactive components is a core mechanism underlying the health effects of foods. Dairy products construct a defense network against oxidation and inflammation through the cooperative actions of multiple constituents. On one hand, antioxidant components in dairy products—such as selenium, vitamin E, and glutathione—scavenge excess reactive oxygen species (ROS) in the body, mitigating oxidative damage to DNA, proteins, and lipids (63, 64). Research by Lee and Han (63) confirmed that vitamin E enhances the body’s antioxidant capacity by modulating the antioxidant enzyme system. On the other hand, components like lactoferrin, CLA and probiotics can inhibit the nuclear factor-kappa B (NF-κB) signaling pathway, thereby reducing the release of pro-inflammatory cytokines and attenuating chronic inflammatory responses (20, 30, 46, 47, 65). Studies indicate that lactoferrin and its derived peptides can exert immunomodulatory effects by inhibiting the NF-κB signaling pathway (65). A study by Habib et al. (66) demonstrated that lactoferrin significantly alleviates inflammation by regulating both the NF-κB and PI3K/Akt pathways. Furthermore, lactoferrin can alleviate tissue inflammatory damage by modulating pathways such as the NLRP3 inflammasome and PI3K/Akt (66).

Antioxidant and anti-inflammatory mechanisms are not independent but exhibit tight synergistic interplay: a reduction in oxidative stress levels can decrease the production of inflammatory factors, while the suppression of inflammatory responses can, in turn, lower the release of ROS, forming a virtuous cycle (20, 64). In a review, Bielecka et al. summarized the antioxidant–anti-inflammatory synergy of dairy protein hydrolysates, confirming that their inhibitory effect on chronic inflammation is significantly superior to that of components with a single mode of action (64). Research by Calder (62) indicated that the linkage between the gut-immune axis and antioxidant–anti-inflammatory mechanisms is a central node in preventing chronic diseases. This synergy plays a key role in preventing metabolic diseases and cancer. For instance, CLA can reduce inflammatory damage to the vascular endothelium through its combined antioxidant–anti-inflammatory effect, thereby lowering the risk of atherosclerosis (30, 67).

### 4.4 Antibacterial and antiviral activities

The antibacterial and antiviral activities of dairy products are primarily achieved through the synergistic actions of bioactive proteins and probiotics (21, 23, 29, 46, 68). Lactoferrin inhibits the proliferation of pathogenic bacteria by chelating iron ions and disrupting bacterial cell membrane integrity (19, 23); a review by Jenssen and Hancock (29) elaborates on the multiple antibacterial mechanisms of lactoferrin, including iron chelation, membrane disruption, and inhibition of bacterial biofilm formation. Lactoperoxidase and lysozyme enhance antibacterial effects by generating antimicrobial substances and disrupting bacterial cell wall structures, respectively (21, 23, 24). Research by Eker et al. (21) confirmed that the lactoperoxidase system exerts significant inhibitory effects on both Gram-positive and Gram-negative bacteria. In terms of antiviral activity, lactoferrin can block the binding of viruses to host cell receptors, thereby inhibiting viral entry, and its hydrolysis product, lactoferricin, shows inhibitory effects against viruses such as SARS-CoV-2 and influenza virus (24, 68).

**Figure 4:**
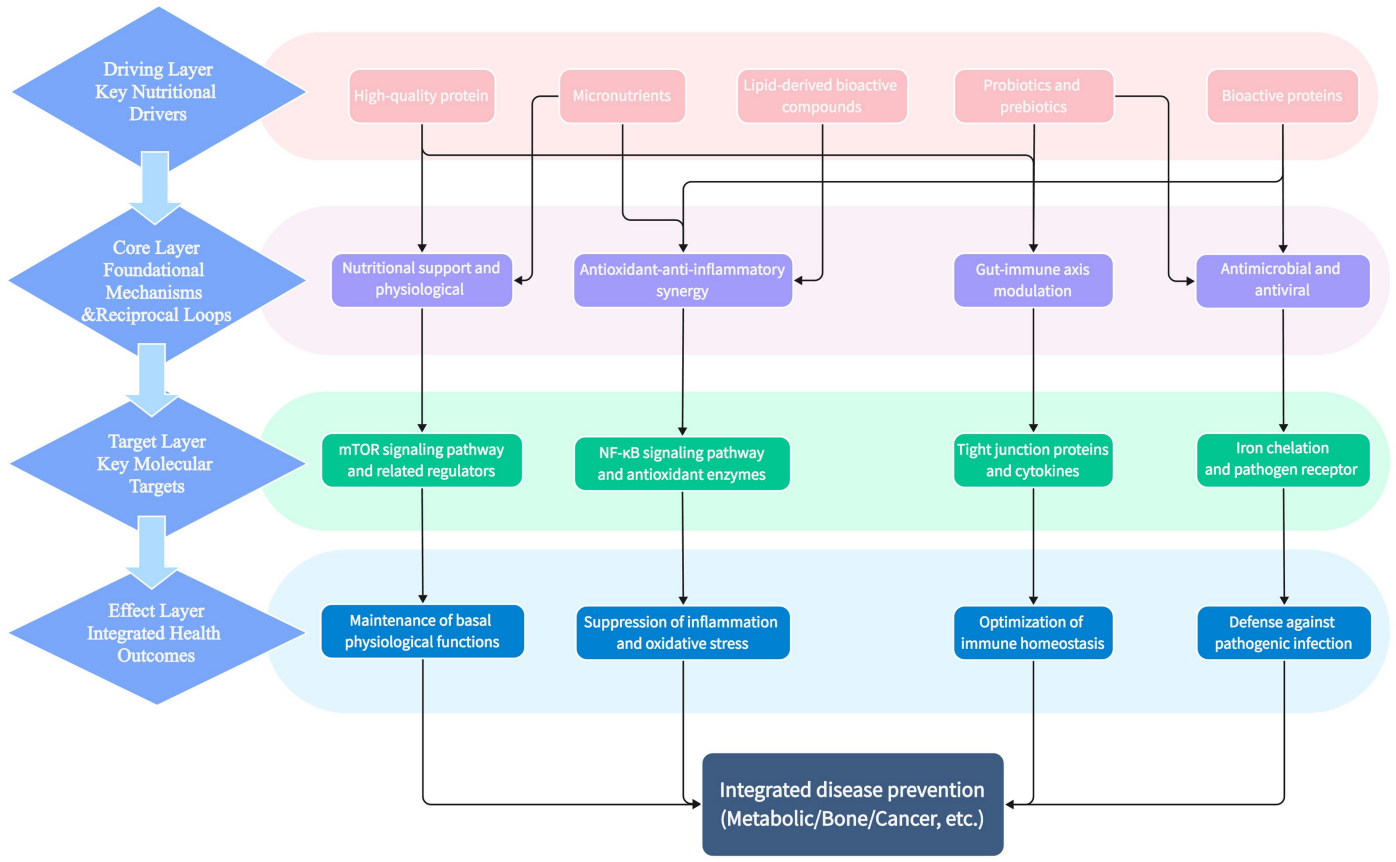
Network diagram of core underlying mechanisms for dairy products exerting health effects. This diagram depicts the interactive regulatory network of the core underlying mechanisms by which dairy products exert health effects, as well as the directional association between core bioactive components, regulatory mechanisms and ultimate health benefits. Nodes represent core components, functional mechanisms and health effect modules; directed lines indicate the regulatory or synergistic relationship between modules.

Probiotics, on the other hand, colonize the intestinal mucosal surface, forming a biological barrier that prevents direct contact between viruses and intestinal epithelial cells, while also modulating the host’s immune response to enhance antiviral capability (46, 47). A study by Vizoso-Pinto et al. (46) confirmed that immunobiotic probiotics can enhance resistance to female genital tract viruses by modulating mucosal antiviral immunity. Furthermore, immunoglobulins in dairy products can directly neutralize viruses, providing the host with passive immune protection (20, 29). Collectively, these mechanisms endow dairy products with potential value in preventing infectious diseases (Figure 4).

## 5 Core preventive effects of dairy products in disease

In summary, dairy products establish an interconnected physiological defense network through multiple fundamental mechanisms, including nutritional support, regulation of the gut-immune axis, antioxidant and anti-inflammatory synergy, and antibacterial/antiviral activities. This network provides a systematic biological explanation for their preventive role in numerous chronic diseases. Following this logic, the subsequent sections will detail the specific effects and supporting evidence of dairy products in the realms of metabolic diseases, bone health, and degenerative as well as malignant diseases.

Building on the aforementioned core fundamental mechanisms, dairy products demonstrate clear preventive value across multiple domains, including metabolic-related diseases, skeletal health, and degenerative and malignant diseases. Notably, their effects are modulated by factors such as total energy intake control, individual metabolic baseline, and the type of dairy product consumed (48, 49, 69–71).

### 5.1 Prevention of metabolic-related diseases

Metabolic-related diseases (including type 2 diabetes, cardiovascular diseases, obesity, etc.) have become major global public health challenges. Dairy products exert comprehensive preventive effects by regulating multiple dimensions such as body weight, glucose metabolism, lipid metabolism, and blood pressure (1–5, 30, 44, 72).

#### 5.1.1 Overview of epidemiological evidence and comprehensive effects

Extensive population studies have provided macro-level evidence for the metabolic protective effects of dairy products. A meta-analysis incorporating multiple prospective cohort studies confirmed that each additional serving of dairy products consumed daily reduces the risk of cardiovascular diseases by 9% and all-cause mortality by 7% (5). The regulatory mechanisms of lipoproteins provide a theoretical basis for understanding how dairy components influence cardiovascular risk (73).

In terms of blood pressure regulation, its positive regulatory effect has been supported by multiple meta-analyses (71, 74-76). The landmark Dietary Approaches to Stop Hypertension (DASH) trial, which included low-fat dairy as a key component, established the efficacy of this dietary pattern in lowering blood pressure (77). A cross-sectional study in a Chinese population also reported an association between higher dairy consumption frequency and lower hypertension prevalence (78). Early reviews have summarized the effects of various natural bioactive substances in milk on the arterial blood pressure system (68). The minerals in dairy products, such as potassium and magnesium, work in synergy with calcium to contribute to blood pressure regulation (79-80). Clinical trials have shown that consuming skim milk enriched with calcium and potassium can elicit favorable blood pressure responses (81). Dairy product consumption can influence intracellular calcium handling and vascular function in individuals with hypertension (82). Specific bioactive peptides (e.g., Val-Pro-Pro) generated during dairy fermentation have been shown to induce acute blood pressure-lowering effects (69, 83, 84). These angiotensin-converting enzyme inhibitory peptides are naturally present in fermented products like cheese (85). Whey protein contains peptides with angiotensin-I-converting enzyme (ACE) inhibitory activity, which is linked to improved vascular health (86).

In terms of body weight regulation, proteins in dairy products can help manage weight by enhancing satiety and reducing total energy intake (87-88). Whey proteins in dairy products have been shown to effectively enhance satiety, aiding in the reduction of subsequent energy intake (87). A further meta-analysis by Abargouei et al. verified a significant dose–response relationship between dairy product intake and weight loss (88). Clinical intervention studies have demonstrated that CLA supplementation can improve body fat percentage and lipid metabolism in obese individuals (72).

Additionally, the consumption of dairy products alongside high-glycemic carbohydrates can modulate postprandial glycemic responses and appetite in older adults (89). These broad health effects suggest that the role of dairy products stems from the systemic regulation of the body’s metabolic network by their multiple components.

#### 5.1.2 Molecular mechanism network of synergistic regulation of glucose and lipid metabolism

**Figure 5:**
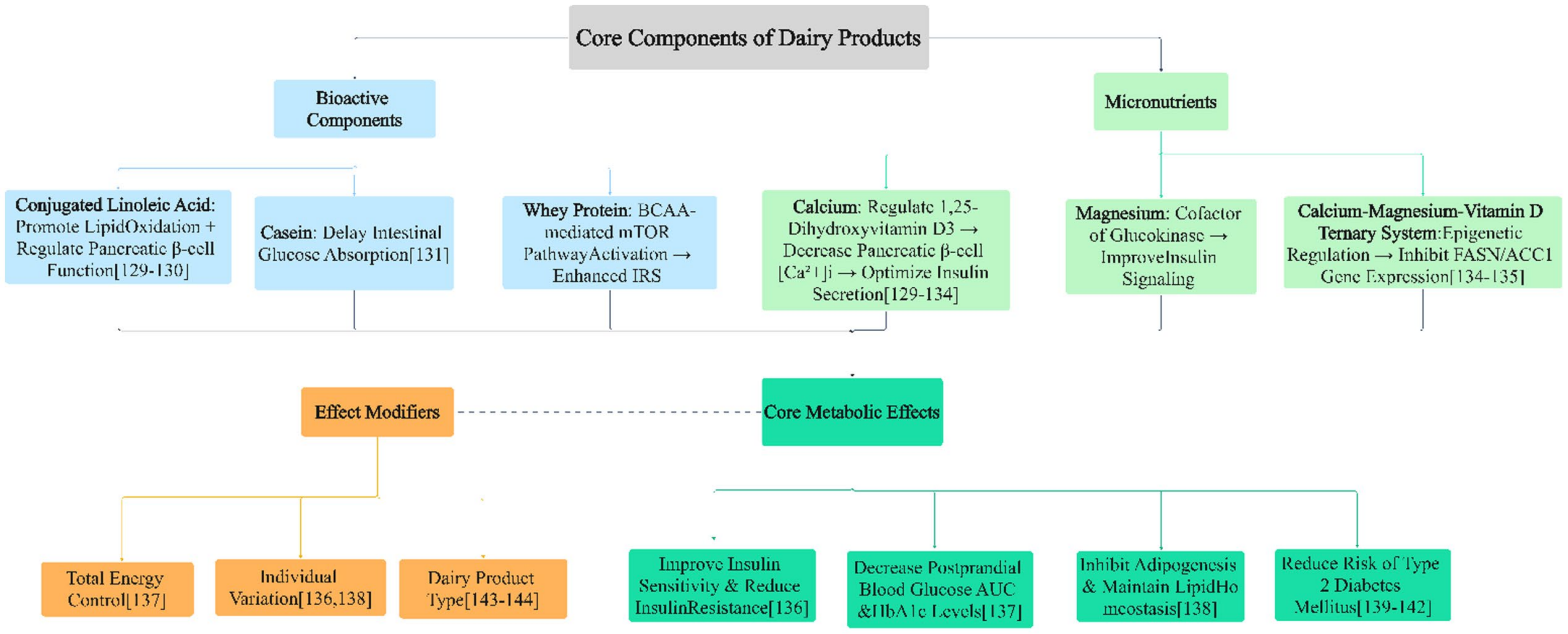
Regulatory mechanism network of dairy product core components on glucose and lipid metabolism and preventive effects on type 2 diabetes. Arrows in the figure represent the regulatory relationship of “component → pathway → effect”; numbers in square brackets correspond to relevant references.

The above macro health effects are rooted in in-depth molecular mechanisms. The prevention of metabolic diseases (such as type 2 diabetes) by dairy products is achieved through a synergistic regulatory network composed of its various core components (see Figure 5), rather than the independent action of a single component.

##### 5.1.2.1 Diversified regulatory pathways of core components

*Lipid metabolism regulation*: CLA activates the PPARα pathway to promote hepatic lipid oxidation, reduce triglycerides, and may regulate pancreatic β-cell function (30–32).

*Glucose homeostasis regulation:* Due to its slow-digestion property, casein helps delay intestinal glucose absorption and stabilize postprandial blood glucose fluctuations (12, 13). Whey protein, with its rich branched-chain amino acids, activates the skeletal muscle mTORC1 signaling pathway to enhance insulin receptor substrate (IRS) phosphorylation, directly improving insulin sensitivity (10, 15, 17).

*Synergy of micronutrients:* The synergistic effect of calcium and vitamin D can effectively optimize the insulin secretion function of pancreatic β-cells (44, 45). As a key cofactor of glucokinase, magnesium is crucial for maintaining the normal function of the insulin signaling pathway (42). Oral magnesium supplementation has been shown to improve endothelial function in patients with coronary artery disease, highlighting its role in metabolic health (90).

##### 5.1.2.2 Integrated effects of the synergistic network

These diversified regulatory pathways intersect with each other, ultimately converging into core effects that improve metabolic health: enhancing insulin sensitivity (17, 44, 45), reducing postprandial blood glucose and glycated hemoglobin (HbA1c) levels (45, 49, 91), and inhibiting adipogenesis while maintaining lipid homeostasis (30–32). Clinical randomized controlled trials provide direct evidence for this: for example, type 2 diabetes patients who consumed vitamin D and calcium-fortified yogurt daily showed a significant reduction in HbA1c levels (45); a high-energy breakfast plan based on whey protein could simultaneously reduce patients’ body weight and HbA1c (49).

##### 5.1.2.3 Factors affecting the effects

It should be noted that the intensity of this regulatory network is modified by multiple factors, including total energy intake, individual basal metabolic status, and the type of dairy product itself (e.g., fermented or not, fat content). It is important to note that some clinical trials have found similar effects on body composition and cardiometabolic health between high-protein and low-protein breakfasts in young overweight women (92). This explains the effect heterogeneity observed in population studies (48, 49, 69–71).

### 5.2 Maintenance of bone and special physiological health

The role of dairy products in maintaining bone health is well-established (93). Components such as calcium, phosphorus, vitamin D, and casein phosphopeptides (CPPs) work synergistically to promote bone mineralization and reduce the risk of osteoporosis and fractures (35, 37, 38, 40). Calcium and phosphorus are present in dairy products in an approximate 2:1 ratio, allowing for their direct incorporation into bone formation (40). Vitamin D promotes intestinal calcium absorption, while CPPs chelate calcium ions, prolonging their residence time in the intestine and further enhancing calcium bioavailability (40, 50). Research by Li et al. (50) confirmed that CPPs can increase intestinal calcium absorption rates by 30%.

For high-risk populations such as older adults and postmenopausal women, moderate daily intake of dairy products is associated with a reduced risk of osteoporosis (36, 39). A cluster randomized controlled trial by Iuliano et al. (37) demonstrated that dietary fortification with dairy products reduced the incidence of hip fractures by 30% among older adult nursing home residents. It is important to note, however, that the association between high dairy intake and fracture risk may be influenced by confounding factors such as overall dietary patterns, leading to some contradictory findings in the literature (94, 95). A cohort study by Michaëlsson et al. (94) found that excessive intake of full-fat dairy products might increase fracture risk in women, whereas low-fat dairy products showed no such effect. This paradox suggests that the relationship between dairy products and bone health is likely not driven by a single component but rather by the complex interplay between their overall nutritional matrix (including fat content, fatty acid profile, calcium bioavailability) and individual metabolic status (such as inflammation levels and hormonal environment). Therefore, future research must move beyond simple associations with “dairy intake quantity” to deeply analyze the specific roles of different dairy types and their components in particular population subgroups. Intervention studies targeting older adult high-risk groups further confirm that synergistic supplementation of dietary calcium and protein significantly reduces the risk of adverse skeletal outcomes (37).

Additionally, dairy products hold significant positive implications for oral health, reproductive endocrine health, and sports medicine (39, 96–99). Their rich content of calcium and phosphorus promotes tooth enamel mineralization, lowering the incidence of dental caries (96). A cohort study by Lempert et al. (96) confirmed that daily dairy intake in children is associated with a 25% reduction in the risk of dental caries. Lactoferrin can inhibit the growth of oral pathogenic bacteria, reducing the occurrence of periodontitis (97). A review by Velliyagounder et al. (97) systematically elaborates on the mechanism of lactoferrin in preventing oral diseases. The cohort study and meta-analysis by Trieu et al. (100) provide more comprehensive evidence-based clarification regarding the association between dairy products and all-cause mortality as well as cardiovascular health.

### 5.3 Prevention of degenerative and malignant diseases

With the intensifying aging of the global population, the need for the prevention and control of degenerative diseases (such as cognitive decline) and malignant diseases (such as breast cancer and colorectal cancer) is becoming increasingly urgent. Dairy products demonstrate potential preventive value through mechanisms such as anti-inflammatory, antioxidant effects, and gut microbiota modulation (32, 34, 101, 102).

Regarding the prevention of cognitive decline, sphingomyelin in the Milk Fat Globule Membrane (MFGM) can promote the formation of neural myelin, thereby maintaining nerve signal conduction function (32, 33); a review by Camfield et al. (34) indicated that MFGM components can improve cognitive function via the gut-brain axis. Tryptophan from whey protein can cross the blood–brain barrier, promoting serotonin synthesis and improving cognitive function and emotional state (103–104); research by Trichia et al. (104) confirmed that gut microbiota metabolites can regulate host serotonin synthesis, influencing cognitive function. Epidemiological studies show a reduced risk of cognitive decline in older adults with daily dairy intake (32, 101); a narrative review by Anderson et al. (101) summarized the evidence for dairy products in preventing cognitive decline, emphasizing their multi-component synergistic effects.

In terms of cancer prevention, bioactive components in dairy products such as CLA and short-chain fatty acids (SCFAs) can exert inhibitory effects on malignancies like breast cancer and colorectal cancer through various mechanisms, including inhibiting tumor cell proliferation, inducing tumor cell apoptosis, and modulating the tumor microenvironment (31, 102, 105, 106). A meta-analysis by Dong et al. (102) confirmed that dairy intake can reduce the risk of breast cancer by 8%. Further research by Aune et al. (105) found that consuming 3 servings of dairy products daily can decrease the risk of colorectal cancer by 15%, with its mechanism potentially closely related to the regulatory effect of SCFAs on gut microbiota and the synergistic antioxidant and anti-inflammatory effects (32, 64, 107).

## 6 Cross-synergistic effects of multiple components and multiple mechanisms

The health effects of dairy products are not the result of the independent action of any single component or mechanism. Instead, they arise from the intersecting synergies among multiple components and multiple mechanisms, forming an integrated regulatory network characterized by “nutritional support → activation of core mechanisms → prevention of multiple diseases.” This constitutes their core advantage over single-nutrient supplements (7, 8, 32).

From the perspective of component synergy, the amino acids provided by high-quality protein serve as the fundamental building blocks for immune cell proliferation, while bioactive proteins such as lactoferrin and immunoglobulins directly modulate immune function. The synergistic action of these two groups enhances the regulatory function of the gut-immune axis (10, 11, 20); a review by Wu et al. emphasized that the synergy between amino acids and bioactive proteins is central to the immunomodulatory effects of dairy products (11). The synergistic action of calcium and vitamin D not only promotes bone mineralization but also indirectly improves glucose metabolism by regulating insulin secretion (38, 39, 90); research by Rizzoli et al. confirmed that the calcium-vitamin D synergy can enhance insulin secretion efficiency by 15% (39). Furthermore, probiotics and prebiotics form a “synbiotic” relationship. The proliferation of probiotics produces short-chain fatty acids (SCFAs), which can synergize with CLA to inhibit intestinal inflammation and tumor cell proliferation (32, 33); concurrently, SCFAs can also exert synergistic effects with other anti-inflammatory components in dairy products, such as lactoferrin (20). Research by Mansilla and Sojo confirmed that the synergy between probiotics and prebiotics can amplify the intestinal anti-inflammatory effect (51). Such synergistic interactions among components enable the health effects of dairy products to far exceed the simple additive effects of individual constituents.

From the perspective of interconnectedness among mechanisms, the optimization of the gut-immune axis can reduce intestinal inflammation and microbial dysbiosis, thereby lowering systemic oxidative stress levels and laying the groundwork for the function of antioxidant and anti-inflammatory mechanisms (20, 49, 63); Calder (62) noted that the linkage between the gut-immune axis and antioxidant-anti-inflammatory mechanisms is key to preventing chronic diseases. Notably, various bioactive components in dairy products coordinately modulate metabolic homeostasis through crosstalk between signaling pathways. Specifically, AMPK, as a core energy sensor, antagonizes the mTORC1 pathway to jointly regulate glycolipid metabolism and autophagy (108, 109). The crosstalk between PPARγ and NF-κB also plays a vital role in improving insulin sensitivity and suppressing chronic low-grade inflammation (110). Additionally, short-chain fatty acids can further regulate these pathways via GPR43/41 receptors, forming a multilevel regulatory network (111). This signaling pathway crosstalk serves as the fundamental molecular basis for the metabolic protective effects of dairy products (108, 110, 111). Furthermore, the synergistic antioxidant and anti-inflammatory effects not only directly inhibit the pathological processes of metabolic diseases and cancer but also protect neural cells and bone tissue, indirectly supporting the prevention of degenerative diseases and the maintenance of skeletal health (31, 32, 67); a review by Bielecka et al. (64) summarized the interconnected effects of antioxidant-anti-inflammatory mechanisms in preventing multiple diseases. Nutritional support, meanwhile, permeates all mechanisms, providing the essential material and energy substrates for gut microbiota balance, immune cell activity, and antioxidant enzyme synthesis, thereby forming a closed loop of “fundamental nutrition → mechanism activation → effect amplification.”

## 7 Research progress and future directions

### 7.1 Latest research progress

In recent years, research on the health effects of dairy products has achieved significant breakthroughs across three dimensions: molecular mechanisms, precision nutrition in populations, and product innovation. At the molecular mechanism level, the application of omics technologies has unveiled novel targets of action for dairy components. For instance, sphingomyelin in the Milk Fat Globule Membrane (MFGM) can improve neural cell synaptic plasticity by regulating the expression of miR-124, providing a new molecular explanation for the prevention of cognitive decline (32, 112); a randomized controlled trial by Ohsawa et al. (112) confirmed that fermented milk containing specific milk-derived peptides can improve cognitive function in middle-aged adults. Whey protein hydrolysate peptides can promote hepatic lipid decomposition by activating the AMPK signaling pathway, and their regulatory mechanism has been validated through integrated transcriptomic and metabolomic analyses (90, 113); a randomized controlled trial by Li et al. (113) confirmed that ACE-inhibitory peptides can lower blood pressure in hypertensive patients by modulating gut microbiota.

In terms of population research, the deepening of the precision nutrition concept has driven the advancement of stratified analyses. Studies targeting individuals with different metabolic phenotypes have found that the glucose metabolism improvement effect of dairy products is significantly greater in insulin-resistant populations (HbA1c reduction of 0.8%) compared to those with normal metabolism (HbA1c reduction of 0.2%) (69, 70, 91); a meta-analysis by Stine et al. (91) confirmed that pre-meal whey protein supplementation has a more pronounced effect on improving postprandial blood glucose in insulin-resistant individuals. Furthermore, a prospective cohort study focusing on the Chinese population confirmed that daily intake of ≥1 serving of low-fat dairy products can reduce the risk of diabetes by 47%, with this effect being more significant among individuals with a BMI < 24 kg/m^2^ (1, 78); the cohort study by Yucheng et al. (1) further validated the inverse association between dairy intake and diabetes risk in the Chinese population. Additionally, the unique health advantages of fermented dairy products are gaining increasing attention. A meta-analysis incorporating 20 randomized controlled trials (RCTs) showed that the preventive effect of fermented dairy products against cardiovascular disease (RR = 0.85) is significantly superior to that of non-fermented dairy products (88, 114). A meta-analysis by Zhang et al. (114) confirmed that consuming fermented dairy products can reduce the risk of cardiovascular disease incidence by 15%. The aforementioned prospective cohort study in the Chinese population indicates that daily intake of ≥1 serving of low-fat dairy products can reduce the risk of diabetes incidence by 47%, and this effect is more pronounced in individuals with a BMI < 24 kg/m^2^ (1).

In the field of product innovation, the development of functional dairy products has become a focal point. Infant formula enriched with lactoferrin can significantly reduce the risk of infectious diarrhea in infants and young children (20, 29); research by Lucky et al. (48) confirmed that buffalo milk-based infant formula can enhance infant immunity. Low-fat yogurt supplemented with conjugated linoleic acid (CLA) and probiotics can reduce body fat percentage by 3.2% in overweight individuals (72, 88); a clinical study by Ye et al. (72) confirmed that CLA-fortified yogurt can improve lipid metabolism in obese individuals. Dairy products fortified with high calcium, vitamin D, and MFGM, targeting older adults, have been shown to improve bone density and cognitive function (32, 90); research by Wada et al. (115) confirmed that functional dairy products can reduce the risk of functional disability and dementia in older adults. Beyond these mainstream products, research has also explored the viability of probiotics in novel food matrices like ice cream for delivering beneficial cultures (116).

### 7.2 Future research directions

While current research has established a fundamental framework for the health effects of dairy products, a complete picture of their complexity and heterogeneity remains to be elucidated. To bridge the gap from “association” to “causation” and “application,” future studies must achieve a paradigm shift across the following four dimensions:

#### 7.2.1 From single pathways to systematic network analysis of mechanisms

Current mechanistic research predominantly focuses on single components or isolated signaling pathways, which falls short of explaining the essence of the synergistic actions among multiple dairy components. Future efforts should leverage spatial multi-omics, single-cell sequencing, and computational biology models to systematically map the dynamic interaction network of “dairy components—gut microbiota—host physiology” at the cellular and animal model levels. The research focus should be on identifying hub molecular targets and regulatory nodes, and clarifying the key mediating role of gut microbiota-derived metabolites (e.g., SCFAs, indole derivatives) within this network. This approach will provide a unified mechanistic explanation at the systems biology level.

#### 7.2.2 From general recommendations to precise stratification in population evidence

Current dietary guideline recommendations are largely based on population averages, overlooking significant individual response variability. There is an urgent need to conduct “biomarker-driven” precision nutrition research.

Precision nutrition strategies based on population heterogeneity represent a promising direction for the application of dairy products. Current dietary guidelines for dairy consumption remain relatively general, and differentiated strategies tailored to individual metabolic characteristics, gut microbiota, and physiological status can further enhance health benefits. (1) For lactose-intolerant individuals, A2 β-casein milk or fermented dairy products are recommended to reduce gastrointestinal discomfort (117); (2) For individuals with hyperlipidemia, dairy products rich in milk fat globule membrane (MFGM) can help regulate cholesterol metabolism and improve lipid profiles (118); (3) For athletic and fitness populations, whey protein supplementation efficiently promotes muscle protein synthesis and post-exercise recovery (119); (4) For middle-aged and older adult individuals at risk of sarcopenia, combined supplementation of whey protein, vitamin D, and calcium helps maintain muscle mass and physical function (119). Precision nutrition strategies based on physiological characteristics and disease risk maximize the health value of dairy products and improve clinical guidance and practical operability (117–119).

By integrating multi-dimensional data (genomic, metabolomic, metagenomic, clinical phenotypes), future studies should define population subgroups that respond differently to the health effects of dairy products. On this basis, stratified randomized controlled trials should be designed to validate whether personalized intake recommendations based on biomarkers can more effectively improve specific health outcomes, thereby laying an empirical foundation for the next generation of precise dietary guidelines.

#### 7.2.3 From phenomenological controversy to causal mechanism clarification

Addressing long-standing controversies, such as those surrounding full-fat dairy products and cardiometabolic risk or high intake and specific fracture risks, requires moving from epidemiological association to mechanistic causal inference. This necessitates conducting prospective cohort and Mendelian randomization studies to control for genetic confounding. Simultaneously, deep phenotyping analyses (e.g., vascular function imaging, bone turnover markers, systemic inflammation indices) should be incorporated into RCTs to reveal the intermediate physiological pathways through which different dairy types produce divergent effects. This approach will transform controversies into a scientific understanding of “dose-component-context” specific relationships.

#### 7.2.4 From functional concepts to evidence-based product translation

Building on the aforementioned mechanistic and population research findings, dairy product innovation should shift from generalized functionality to targeted design for disease prevention. The core lies in developing evidence-based functional products for specific risk stages (e.g., prediabetes, high-risk period for osteoporosis). This relies on: ① Bioactive stabilization technologies (e.g., microencapsulation, directed fermentation) to ensure functional preservation during processing, storage, and transportation; and ② Clinical endpoint validation, where the improvement of clinical hard endpoints (e.g., diabetes incidence, fracture rate) is confirmed through rigorous RCTs, thereby realizing the translation of value from nutritional science to public health products.

Future research on dairy nutrition and health should further focus on the nutritional needs of special populations, including pregnant women, infants, older adults individuals with sarcopenia, postoperative rehabilitation patients, and patients with chronic diseases, and explore the metabolic interactions of dairy products with other food components under different dietary patterns (e.g., Mediterranean diet, high-protein diet, plant-based diet). Through high-quality cohort studies, precision nutrition intervention trials, and molecular mechanism analyses, basic research results can be translated into individualized dietary guidelines, clinical nutrition programs, and public health policies, providing high-level evidence for the scientific application of dairy products in chronic disease prevention and health promotion (120–121).

## 8 Conclusion

Dairy products are far more than simple sources of nutrition; they are, in essence, a complex nutritional matrix with high bioactivity. This review elucidates that their health value stems from the precise integration and synergistic actions of multiple functional components (such as high-quality protein, lactoferrin, CLA, calcium-vitamin D complexes, and probiotics) through a multitude of mechanisms, including nutritional support, gut-immune axis regulation, and antioxidant-anti-inflammatory synergy (7, 8). This integrated model of “multiple components—multiple mechanisms—multiple effects” constitutes the scientific basis for their demonstrably positive impact on preventing metabolic diseases (e.g., type 2 diabetes, cardiovascular diseases), maintaining skeletal health, and potentially reducing the risk of certain degenerative and malignant diseases (1–6, 32, 46, 100, 103).

However, the health effects of dairy products exhibit significant population heterogeneity and context-dependence. Individual metabolic baselines, overall energy balance, and, in particular, the type of dairy product (e.g., fermented vs. non-fermented, fat content) serve as key modifying factors that collectively determine the direction and magnitude of the net health effect (48, 49, 69, 70). This complexity indicates that future research must move beyond the realm of “average effects,” striving to elucidate the biological basis of this heterogeneity and thereby drive precision nutrition practices.

Based on current epidemiological and clinical intervention evidence, incorporating moderate amounts of dairy products (1–2 servings daily, prioritizing fermented or low-fat options) into a balanced dietary pattern represents a reasonable public health strategy for reducing chronic disease risk and promoting health across the lifespan (86, 112). Looking forward, by deepening systems biology-based mechanistic, conducting prospective stratified intervention studies, and promoting the development of evidence-based functional products, we can aspire to translate the health potential of dairy products into more precise and personalized clinical and public health practices. This endeavor holds promise for playing an even greater role in alleviating the global burden of disease.

## Data Availability

Publicly available datasets were analyzed in this study. This data can be found at: This is a systematic review article. All data synthesized and analyzed are derived from the previously published studies cited in the reference list. Therefore, a direct link to a specific dataset or repository accession number is not applicable.
